# Smoking status and tumor necrosis factor-alpha mediated systemic inflammation in COPD patients

**DOI:** 10.1186/1476-9255-7-29

**Published:** 2010-06-09

**Authors:** Suzana E Tanni, Nilva RG Pelegrino, Aparecida YO Angeleli, Camila Correa, Irma Godoy

**Affiliations:** 1 Department of Internal Medicine, Pulmonology Division, Botucatu Medical School. UNESP (Paulista State University). Distrito de Rubião Júnior, s/n. Botucatu, 18618-000, SP, Brazil; 2 Department of Internal Medicine, Botucatu Medical School. UNESP (Paulista State University). Distrito de Rubião Júnior, s/n. Botucatu, 18618-000, SP, Brazil

## Abstract

**Background:**

Smoking cause airway and systemic inflammation and COPD patients present low grade inflammation in peripheral blood. However, data on the influence of smoking itself on systemic inflammation in COPD patients are scarce. This study investigated the association between inflammation, smoking status, and disease.

**Methods:**

A cross-sectional analysis comparing 53 COPD ex-smokers, 24 COPD current smokers, 24 current smoker controls and 34 never-smoker controls was performed. Assessments included medical history, body composition, spirometry, and plasma concentration of tumor necrosis factor-alpha (TNF-α), interleukins (IL)-6, IL-8, and C-reactive protein (CRP).

**Results:**

Our exploratory analysis showed that serum TNF-α was higher in COPD current smokers [4.8(4.2-5.8)pg/mL] and in current smoker controls [4.8 (4.2-6.1) pg/mL] when compared to COPD ex-smokers [4.3 (3.9-4.9)pg/mL; p = 0.02] and to never-smoker controls [3.7 (3.4-4.0)pg/mL; p < 0.001]. Multiple regression results with and without adjustment for covariates were consistent with the hypothesis that TNF-α levels were associated with smoking status in both models (p < 0.001 and p < 0.001). IL-6 and CRP were significantly higher in COPD patients when compared to smoker and never-smoker controls and the multiple regression analysis confirmed the association of these mediators with disease, but not with smoking status (p < 0.001 and p < 0.001). IL-8 had only a borderline association with disease in both models (p = 0.069 and p = 0.053). No influence of disease severity, inhaled corticosteroid, fat-free mass (FFM) depletion and long term oxygen therapy (LTOT) use on systemic inflammation was found.

**Conclusion:**

Smoking may influence TNF-α mediated systemic inflammation, which, in turn, may account for some of the benefits observed in patients with COPD who stop smoking.

## Background

Cigarette smoking is a major risk factor for chronic obstructive pulmonary disease (COPD). Long-term smoking causes airway inflammation characterized by neutrophil, macrophage, and activated T lymphocyte infiltration and by increased cytokine concentrations such as tumor necrosis factor-alpha (TNF-α), interleukins (IL)-6 and IL-8 [1-4]. Although nearly all smokers show some evidence of lung and systemic cellular and/or humoral inflammation, only a few will suffer an amplified response and develop COPD [5,6].

Several studies have shown systemic inflammation in COPD patients with increased neutrophil, macrophage, and T-lymphocyte numbers and high concentrations of inflammatory mediators in peripheral blood (C-reactive protein (CRP), IL-6, IL-8 and TNF-α) [7-12]. TNF-α, a powerful pro-inflammatory cytokine primarily produced by activated macrophages, is thought to play a critical role in the pathogenesis of COPD by promoting and maintaining the expression and release of various proinflammatory mediators which lead to tissue damage and remodeling [13,14].

Very little is known about the mechanism of increased TNF-α concentration in the plasma of COPD patients and its relationship with disease severity and active smoking has not been established [9,15]. Evaluation of soluble receptors, an indirect marker of proinflammatory state related to systemic TNF-α, showed no influence of smoking on systemic inflammation in small sample of COPD patients [16]. However, in a study with the aim to examine levels of inflammatory markers in COPD and asthma patients, the influence of smoking on TNF-α serum concentration was identified only in the subgroup of COPD patients [12]. We hypothesed that active smoking may be associated with more severe systemic inflammation in COPD patients. In order to test our hypothesis, we analyzed concentrations of TNF-α, IL-6, IL-8 and CRP in the peripheral blood of current smoker and ex-smoker COPD patients, with a wide range of airway, current smoker and never-smoker controls.

## Methods

### Subjects

Seventy seven clinically stable COPD (mild to very severe) patients, 53 ex-smokers (for at least 1 year) and 24 current smokers, followed-up at the Outpatient Pulmonology Unit of Botucatu Medical School - Brazil participated in the study. COPD was diagnosed according to the GOLD criteria: post-bronchodilator (400 mcg fenoterol) FEV_1_/FVC ratio < 70% without significant reversibility (<11% predicted FEV_1 _or 200 ml) in the presence of respiratory symptoms and a smoking history of at least 20 pack-years [17]. Exclusion criteria included oral steroid use or exacerbations in the last three months before enrollment in the study, diagnosis of other chronic diseases (i.e. diabetes, renal failure and cancer) or other respiratory disease (i.e. asthma, bronchiectasis, bronchiolitis or tuberculosis). Further 24 current smoker controls (at least 10 pack-years) with no evidence of COPD, other diseases or using regular medication and 34 never-smoker controls were also recruited. Smokers and never-smoker controls underwent routine clinical assessments including spirometry and chest X-ray. Ethical approval was obtained from Ethical Committee of Botucatu Medical School - Brazil and all subjects gave written informed consent.

### Pulmonary function test and peripheral oxygen saturation

Forced expiratory volume in the first second (FEV_1_) and forced vital capacity (FVC) were obtained from the flow-volume curve using a spirometer (Ferraris KOKO, Louisville, CO, USA) before and 20 minutes after β-agonist (fenoterol 400 mcg) inhalation. The highest value of at least three measurements was selected and expressed as a percentage of reference values [18]. Pulse oximetry (SpO_2_%) was assessed by a portable oxymeter (Nonin Medical, Plymouth, MN, USA).

### Body composition

Height and weight were measured and body mass index (BMI) calculated (kg/m^2^). Fat-free mass (FFM), in kg, was estimated using bioelectrical impedance analysis (BIA 101, RJL systems, Detroit, MI, USA) according to European Society Parenteral Enteral Nutrition guidelines [19] using specific equations for COPD patients and healthy controls [20,21]. FFM index (FFMI) was calculated as FFM/height^2 ^(kg/m^2^). FFM depletion was diagnosed when FFMI < 15 kg/m^2 ^for females and <16 kg/m^2 ^for males was present [22].

### Blood sampling and analysis

Fasting peripheral blood was collected early morning (08.00-10.00 hours) and plasma stored at -80°C until analysis. TNF-α, IL-6, and IL-8 were assessed in duplicate by high sensitivity commercial kits using enzyme linked immunosorbent assay (ELISA) according to manufacturer's instructions (BioSource International Inc, Ca, USA). Lower detection limit was 0.5 pg/mL for TNF-α, 0.16 pg/mL for IL-6, and 0.39 pg/mL for IL-8. C-reactive protein (CRP) was assessed in duplicate by high sensitivity particle enhanced immunonephelometry (CardioPhase, Dade Behring Marburg GmbH, Marburg, USA) with a lower detection limit of 0.007 mg/L.

### Statistical analysis

All variables were summarized using means and standard deviations. To compare groups defined by smoking status and presence of disease (COPD) on demographic and general characteristics we used Chi-square tests for categorical variables and analysis of variance (ANOVA) for continuous variables, using the Tukey test to account for multiple comparisons. Kruskal-Wallis tests were used to evaluate group differences for the inflammation measures with Dunn's method used to adjust for multiple comparisons [23]. We analyzed the association of smoking status (current smoking or no-smoking) and presence of disease (COPD) with systemic inflammation (TNF-α, IL-6, IL-8 and CRP) using a multiple linear regression with robust standard errors with and without adjustment for potential confounders (age, gender, SpO_2 _and FFM). Statistical significance was considered when p < 0.05. (STATA software 9.0 - STATA Corp, College Station, Texas, USA).

## Results

### Populations Characteristics

Demographics, baseline lung function, and body composition of never-smoker and current smoker controls, COPD ex-smokers and COPD current smokers are presented in Table 1. COPD patients tended to be older than current smoker controls and never-smoker controls; smoking history (pack-years) was similar in COPD patients and current smoker controls. As expected, COPD patients had significant airflow limitation and decreased SpO_2 _compared to never-smoker and current smoker controls. Twelve COPD patients were at GOLD stage I, 28 GOLD stage II, 14 GOLD stage III, and 23 GOLD stage IV. Sixty five patients were using long-term broncodilators and seventeen (tree COPD current smokers and 14 ex-smokers) patients were regularly using inhaled corticosteroid (800 mcg of budesonide), 16 had been on stable oxygen flow therapy for the last six months. No patients were medicated with theophylline or leukotriene modifiers.

**Table 1:** Demographics, lung function, and body composition of never-smoker controls and current smoker controls and COPD patients, according to smoking status

	Never-smoker controls N = 34	Current smokers controls N = 24	COPD ex-smokers N = 53	COPD Current smokers N = 24	p value
Age (years)	49.1 ± 8.3	48.6 ± 6.6	66.5 ± 7.7*	57.5 ± 9.3^††^	<0.001
Gender (M/F%)	50/50	58/42	75/25^§^	46/54^§^	0.03
FEV_1 _(L)	3.3 ± 0.7	2.9 ± 0.8	1.3 ± 0.7*	1.5 ± 0.9*	<0.05
FEV_1 _(%)	111.2 ± 15.2	105.4 ± 16.2	56.3 ± 25.5*	59.4 ± 22.1*	<0.001
FVC (L)	4.0 ± 0.9	3.8 ± 0.8	2.7 ± 0.8*	2.7 ± 1.1*	<0.05
FVC (%)	110.8 ± 14.4	111.6 ± 16.9	90.8 ± 24.1*	89.5 ± 19.9*	<0.001
FEV_1_/FVC (%)	83.2 ± 14.8	78.0 ± 4.9	49.0 ± 12.8*	53.5 ± 12.1*	<0.05
Packs-year	0.0(0.0-0.0)	30.0(21.3-40.0)^†^	50.0(29.1-62.5)^†^	44.0(32,0-60.0)^†^	<0.05
SpO_2 _(%)	96.6 ± 1.4	96.5 ± 1.5	91.2 ± 5.5*	93.4 ± 3.6*	<0.05
BMI (kg/m^2^)	25.5 ± 3.3	23.8 ± 3.9	25.4 ± 5.5	25.1 ± 4.7	0.48
FFM (kg)	46.3 ± 8.6	44.6 ± 7.4	42.0 ± 7.4	38.3 ± 7.1^†^	0.01
FFMI (kg/m^2^)	17.3 ± 1.8	16.5 ± 1.9	16.1 ± 2.4	15.3 ± 1.9^†^	0.005

Values are expressed as mean (SD) or median (range).

Differences between groups were tested using one way analysis of variance (ANOVA) followed by the Tukey test to account for multiple comparisons. Differences between proportions were tested using Chi-square.

* compared to never-smoker controls and current smokers controls. ^† ^compared to never-smoker controls. ^†† ^compared to never-smoker controls, current smoker controls and ex-smokers COPD. ^§ ^difference in proportion of gender within COPD groups.

M/F: male/female, FVC: forced vital capacity, FEV_1_: forced expiratory volume in the first second, SpO_2_: percentage arterial oxygen saturation, BMI: body mass index, FFM: fat free mass, FFMI: fat free mass index.

FFM depletion was seen in COPD patients compared to never-smoker controls (Table 1). Five (14.7%) never-smokers, four (16.7%) current smoker controls and thirty seven (48.1%) COPD patients showed FFM depletion; however, the prevalence was not statistically different between groups (p = 0.46).

### Plasma levels of TNF-α, IL-6, IL-8, and CRP

Plasma TNF-α was higher in current smoker controls and COPD current smokers when compared to never-smoker controls, and COPD ex-smokers (Table 2). The regression results, both, with and without adjustment for age, gender, SpO_2 _and FFM also showed a positive association between active smoking and TNF-α (Table 3). We did not found difference in serum TNF-α between the groups according the use of inhaled corticosteroid (p = 0.49).

**Table 2:** Systemic inflammation in never-smoker controls, current smokers controls, COPD ex-smokers and COPD current smokers

	Never-smoker controls N = 34	Current smoker controls N = 24	COPD ex-smokers N = 53	COPD current smokers N = 24
TNF-α(pg/mL)	3.7 (3.4-4.0)	4.8 (4.2-6.1) *	4.3 (3.9-4.9) ^‡^	4.8(4.2-5.8) *
IL-6 (pg/mL)	0.35 (0.21-0,44)	0.42 (0.29-0.81)	0.88 (0.52-1.41) ^‡†^	0.83(0.46-1.33) ^‡^
IL-8 (pg/mL)	5.47 (4.45-7.85)	5.68 (4.32-8.35)	5.53 (3.87-11.82)	5.77(3.87-10.53)
CRP (pg/L)	1.10 (0.62-2.00)	1.07 (0.56-2.54)	4.65 (1.62-7.92) ^‡†^	6.85(2.97-9.39)^‡†^

TNF-α:Tumor necrosis factor alpha, IL-6: interleukin 6, IL-8: interleukin 8, CRP: C-reative protein. Kruskal-Wallis test followed by Dunn's Method to account for multiple comparisons.

* p < 0.05 when compared to never-smoker controls and COPD ex-smokers. ^‡ ^p < 0.05 when compared to never-smoker controls. ^† ^p < 0.05 when compared to current smoker controls.

**Table 3:** Regression models with robust standard errors for TNF-α, IL-6, IL-8 and CRP

Model	Unadjusted (95% confidence interval)	Adjusted (95% coefidence interval)
**TNF-α(pg/mL)**		
Current smoking status (yes)	1.032 (0.592,1.471)	1.224 (0.724,1.724)
Presence of disease (COPD) (yes)	0.323 (-0.049,0.696)	0.110 (-0.457,0.677)
Age (years)	--	0.018 (-0.009,0.415)
Gender (Male)	--	0.0005 (-0.6048,0.6049)
SpO_2 _(%)	--	-0.018 (-0.056,0.019)
FFM (kg)	--	0.028 (-0.008,0.066)

**IL-6 (pg/mL)**		
Current smoking status (yes)	0.110 (-0.183,0.404)	0.185 (-0.177,0.547)
Presence of disease (COPD) (yes)	0.621 (0.382,0.861)	0.270 (-0.028,0.569)
Age (years)	--	0.019 (-0.001,0.038)
Gender (Male)	--	-0.022 (-0.329,0.284)
SpO_2 _(%)	--	-0.022 (-0.065,0.021)
FFM (kg)	--	-0.004 (-0.029,0.020)

**CRP (mg/L)**		
Current smoking status (yes)	0.893 (-2.379,4.165)	2.447 (-1.025,5.918)
Presence of disease (COPD) (yes)	6.834 (3.673,9.994)	4.939 (0.958,8.920)
Age (years)	--	0.175 (0.006,0.344)
Gender (Male)	--	-0.494 (-3.361,2.372)
SpO_2 _(%)	--	0.028 (-0.433,0.488)
FFM (kg)	--	0.141 (-0.018,0.302)

**IL-8 (pg/mL)**		
Current smoking status (yes)	-0.829 (-13.738,12.080)	-3.836 (-19.490,11.818)
Presence of disease (COPD) (yes)	11.479 (-0.889,23.847)	24.361 (-0.287,49.009)
Age (years)	--	-0.516 (-1.272,0.240)
Gender (Male)	--	-4.121 (-21.129,12.888)
SpO_2 _(%)	--	1.205 (-0.842,3.254)
FFM (kg)	--	0.831 (-2.633,0.799)

TNF-α: tumor necrosis factor-alpha; IL-6: interleukin 6; IL-8: interleukin 8; CRP: C-reactive protein; SpO_2_: peripheral saturation; FFM: fat free mass.

Multiple regression with robust standard erros for the analysis of association of current smoking status and presence (COPD) on systemic inflammation (TNF-α, IL-6, CRP and IL-8). The first column shows models without adjustment and the second column shows models with adjustment.

Plasma CRP was higher in both groups of COPD patients when compared to never-smoker and current smoker controls. IL-6 also was higher in both groups of COPD patients when compared to never-smoker controls and also was higher in COPD ex-smokers when compared to current smoker controls (Table 2). Unadjusted regression results also showed an association between CRP, IL-6 and the presence of the COPD; however, no association with active smoking was detected. IL-8 had only a borderline association with COPD in both models (p = 0.069 and p = 0.053) (Table 3).

We compared systemic inflammation in COPD patients according to GOLD stage, presence of nutritional depletion (FFMI), and long term oxygen therapy (LTOT) and no difference was found between groups (data not shown).

## Discussion

This study aimed to evaluate the relationship between systemic inflammation and smoking status in COPD patients. The main finding was that current smokers presented significantly higher plasma levels of TNF-α compared to no-smokers. These results suggest that smoking may be associated with higher TNF-α mediated systemic inflammation in COPD patients who are current smokers. We also reinforce previous findings that patients with COPD, regardless of smoking status, present evidence of systemic inflammation when compared to control subjects.

We could not identified studies designed to assess the influence of active smoking on plasma TNF-α concentration in COPD patients. Higashimoto et al. showed increased levels of serum TNF-α, IL-6 and tissue inhibitors of metalloproteinase-1 in asthma and COPD patients when compared to control subjects. In the subgroups analysis, in partial agreement with our findings, they showed increased levels of TNF-α in current smoker with COPD when compared to ex-smoker COPD patients; however, they failed to show higher levels of TNF-α in current smoker control subjects when compared to non-current smoker control subjects [12]. Vernooy et al. compared local and systemic inflammation in a small sample of COPD patients (ex-smokers = 6; current smokers = 12) and did not show influence of smoking on plasma concentration of soluble TNF receptors (sTNF-R55 and sTNF-R75) [16]. Although the TNF receptors are considered to be markers of a proinflammatory state inducible by cytokines such as TNF-α, they present different biological functions [24,25]. In summary, our results bring new evidence related to the influence of active smoking on TNF-α plasma concentration in control subjects and COPD patients.

There is consensus about the presence of small airway and lung parenchyma inflammation in smokers and COPD patients [3,4,16,26-28]. Local inflammation is characterized by increased numbers of inflammatory cells, such as neutrophils, lymphocytes, and macrophages and higher TNF-α and IL-8 concentrations than healthy controls [1,3,4,26,27,29-32]. However, it is unclear whether established inflammation in smokers returns to normal after smoking cessation [2,33]. In a recent study, Gamble et al. 2007 compared bronchial biopsies of COPD current smokers and COPD ex-smokers and did not find any differences in cell counts or inflammatory markers (CD8+, CD4+, CD 68+, or TNF-α) between groups [2]. In another study, neutrophil and lymphocyte numbers, and IL-8 concentration in the sputum of COPD patients increased one year after smoking cessation [33].

Smoking cessation is the only management measure associated with reduced FEV_1 _decline in COPD patients [5,17]; however, to date, no reduction in airway inflammation has been demonstrated when ex-smokers are compared to current smokers [2,33]. We speculate that the association of smoking with systemic TNF-α concentrations observed in this study may partially explain some of the benefit associated with smoking cessation [17,34,35].

Our results showed higher serum CRP and IL-6 levels in COPD patients than in never-smoker and current smoker controls. Several previous studies have also shown higher serum CRP levels in COPD patients than in no-smoking controls and current smokers [8,11,36]. Higher CRP concentration has been associated with airway obstruction severity, increased resting energy expenditure, and impaired exercise capacity and quality of life in COPD patients [15,37]. Although there is little data regarding IL-6 concentration in COPD patients [8,9], in agreement with our findings elevated serum IL-6 levels have been shown in COPD patients compared to no-smoker controls [15,36,38].

No influence of airway obstruction severity and use of LTOT on systemic inflammation in COPD patients was observed in our study. This result is in contrast with findings of a recent report showing higher serum TNF-α levels in COPD patients with FEV_1 _< 30% in comparison with mild-moderate COPD patients; however, the comparison between groups was not adjusted for smoking status of the patients [40]. In agreement with previous findings no difference in inflammatory markers was also shown when COPD patients with and without FFM depletion were compared [39,40]. In addition, we did not show influence of inhaled corticosteroids on systemic inflammation in COPD patients. However, when we classified the COPD patients according GOLD classes, use of LTOT or inhaled corticosteroids we had few patients to compare between groups and poor power to detect potential differences.

There were other limitations to this study. Firstly, the analyses were performed using cross sectional data and therefore valid inferences regarding causal pathways cannot be drawn. Secondly, we did not analyze local inflammation and some data suggest that local and systemic inflammation can be regulated differently [16].

## Conclusions

In conclusion, smoking is associated with higher levels of TNF-alpha mediated systemic inflammation in patients with or without COPD. In patients with COPD, who already have elevated levels of TNF-alpha, active smoking may associated with even higher degree of the systemic inflammation. Longitudinal studies evaluating the effects of smoking cessation on bronchial and systemic inflammation are needed to allow better understanding of these relationships and their consequences.

## Abbreviations

BMI: Body Mass Index; COPD: Chronic Obstructive Pulmonary Disease; CRP: C- Reactive Protein; FEV_1_: Forced Expiratory Volume in one second; FEV_1_/FVC: Forced Expiratory Volume in one second/Forced Vital Capacity; FFM: Fat- Free Mass; FFMI: Fat-Free Mass Index; FVC: Forced Vital Capacity; GOLD: Global Initiative for Chronic Obstructive Lung Disease; IL: Interleukin; Ln: Natural logarithm; LTOT: Long-Term Oxygen Therapy; SpO_2_: Peripheral Oxygen Saturation; TNF-α: Tumor Necrosis Factor alpha.

## Competing interests

The authors declare that they have no competing interests.

## Authors' contributions

The authors' responsibilities were as follow. SET: performed selection and the medical assessment of the individuals, statistical analysis and interpret the data and draft the final manuscript; NRGP: performed the medical assessment; AYOA: conducted the laboratory analysis; CRC: laboratory analysis; IG: had overall responsibility for the study, designed the research, analyzed and interpret the data, and wrote the final manuscript. All the authors contributed to the revision of the manuscript.
